# Who Should Make Care Arrangement for Older Adults? Heteronormative Family Responsibility in Japan

**DOI:** 10.1093/geroni/igab046.1628

**Published:** 2021-12-17

**Authors:** Ryo Hirayama

**Affiliations:** Osaka City University, Osaka, Osaka, Japan

## Abstract

In Japan, despite the greater availability of public care services upon implementation of national long-term care insurance, families are still considered as primarily responsible to make care arrangement for older adults. My aim in this study was to explore (hetero)normative ideas about families that underlie Japan’s institutionalized practices of elder care. In doing so, I focused on care managers, who are certified care practitioners helping families to make care arrangement, and whether they would count older adults’ same-sex partners as legitimate family members to participate in such arrangement. Data were collected from 1,580 care managers working for officially designated in-home care providers across the nation. Preliminary analysis revealed that although most care managers believed the voices of same-sex partners should be preferably reflected in the process of care arrangement, they also thought that these partners could not participate in such process without permission from older adult’s “blood relatives” (e.g., siblings).

